# Supplementary material for: Garvicins AG1 and AG2: Two Novel Class IId Bacteriocins of Lactococcus garvieae Lg-Granada
Source: Int J Mol Sci. 2022 Apr 23;23(9):4685. doi: 10.3390/ijms23094685 (PMC9101539; doi:10.3390/ijms23094685)
Supplement: Supplementary file 1 [file ijms-23-04685-s001.zip › ijms-1679106-supplementary.pdf]

A

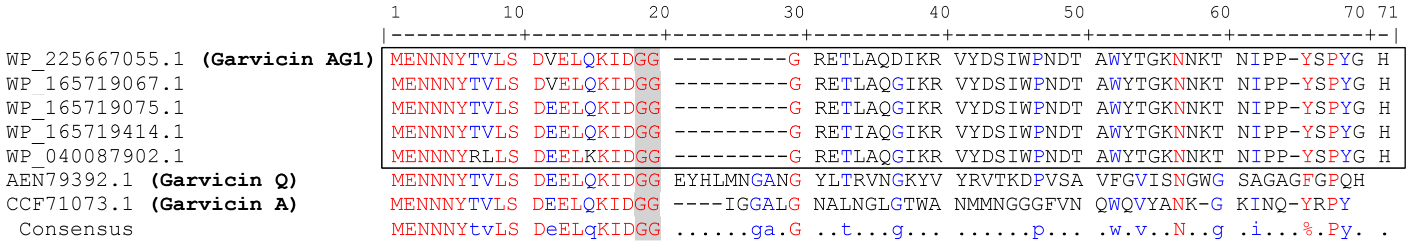
1 10 20 30 40 50 60 70 71
WP_225667055.1 (Garvicin AG1) MENNNYTVLS DVELQKIDGG ---------G RETLAQDIKR VYDSIWPNDT AWYTGKNNKT NIPP-YSPYG H
WP_165719067.1 MENNNYTVLS DVELQKIDGG ---------G RETLAQGIKR VYDSIWPNDT AWYTGKNNKT NIPP-YSPYG H
WP_165719075.1 MENNNYTVLS DEELQKIDGG ---------G RETLAQGIKR VYDSIWPNDT AWYTGKNNKT NIPP-YSPYG H
WP_165719414.1 MENNNYTVLS DEELQKIDGG ---------G RETIAQGIKR VYDSIWPNDT AWYTGKNNKT NIPP-YSPYG H
WP_040087902.1 MENNNYRLLS DEELKKIDGG ---------G RETLAQGIKR VYDSIWPNDT AWYTGKNNKT NIPP-YSPYG H
AEN79392.1 (Garvicin Q) MENNNYTVLS DEELQKIDGG EYHLMNGANG YLTRVNGKYV YRVTKDPVSA VFGVISNGWG SAGAGFGPQH
CCF71073.1 (Garvicin A) MENNNYTVLS DEELQKIDGG ----IGGALG NALNGLGTWA NMMNGGGFVN QWQVYANK-G KINQ-YRPY
Consensus MENNNYtvLS DeELqKIDGG ......ga.G ..t...g... ......p... .w.v..N..g .i...%.Py. .

B

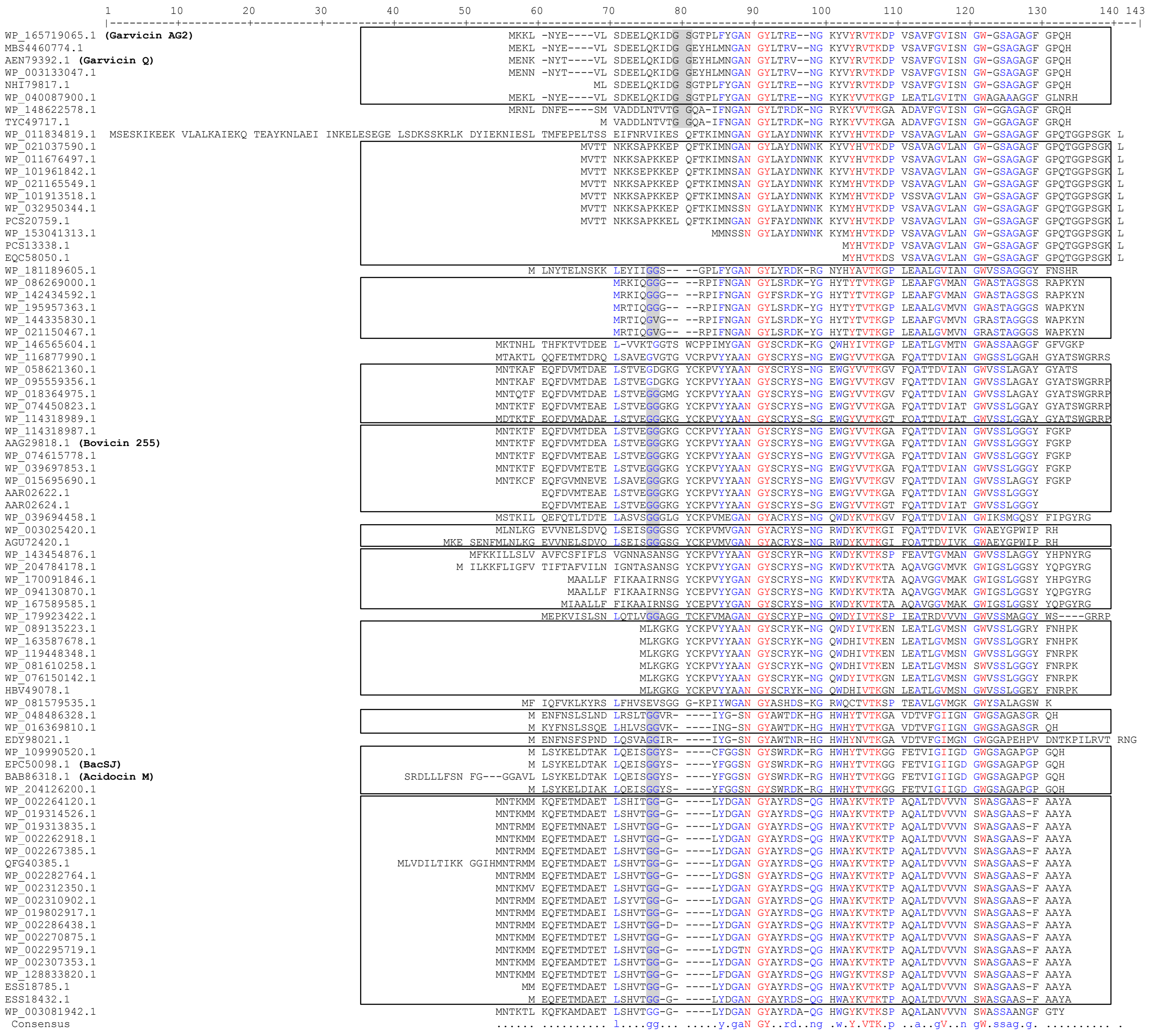

Supplementary Figure 1 (Figure S1). Alignment of the amino acid sequences of Garvicin AG1 (A) and Garvicin AG2 precursor peptides (B) with its closest homologs (determined by Protein Blast). The double-glycine motifs (GG, GA or GS) of the leader peptides are highlighted in grey background. The sequences were aligned at the Multalin interface page (Florence Corpet – INRA). Highly conserved residues (consensus level=90%) are in red, while weakly conserved ones (consensus level=50%) are in blue. Consensus symbols are: !, I or V; $, L or M; %, F or Y; #, N, D, Q, E, B or Z. Boxes mark groups of identical or highly similar related peptides.

NCBI Reference Sequences (non-redundant protein sequence) or GenBank Accession numbers are:
WP_225667055.1 (**Garvicin AG1**), hypothetical protein (plasmid) [*Lactococcus garvieae* Lg-Granada]; WP_165719065.1 (**Garvicin AG2**), garvicin Q family class II bacteriocin (plasmid) [*Lactococcus garvieae* Lg-Granada]; WP_165719067.1, hypothetical protein [*Lactococcus petauri*]; WP_165719075.1, MULTISPECIES: bacteriocin [*Lactococcus*]; WP_165719414.1, bacteriocin [*Lactococcus petauri*]; WP_040087902.1, MULTISPECIES: hypothetical protein [*Lactococcus*]; AEN79392.1 (**Garvicin Q**), prepeptide GarQ (plasmid) [*Lactococcus garvieae*]; CCF71073.1 (**Garvicin A**), hypothetical protein (plasmid) [*Lactococcus garvieae*]; MBS4460774.1, class II bacteriocin garvicin Q [*Lactococcus petauri*]; WP_003133047.1, class II bacteriocin garvicin Q [*Lactococcus garvieae*]; NHI79817.1, bacteriocin [*Lactococcus petauri*]; WP_040087900.1, garvicin Q family class II bacteriocin [*Lactococcus garvieae*]; WP_148622578.1, garvicin Q family class II bacteriocin [*Weissella muntiaci*]; TYC49717.1, bacteriocin [*Weissella muntiaci*];WP_011834819.1, MULTISPECIES: garvicin Q family class II bacteriocin [*Lactococcus*]; WP_021037590.1, garvicin Q family class II bacteriocin [*Lactococcus cremoris*]; WP_011676497.1, garvicin Q family class II bacteriocin [*Lactococcus cremoris*]; WP_101961842.1, garvicin Q family class II bacteriocin [*Lactococcus lactis*]; WP_021165549.1, MULTISPECIES: garvicin Q family class II bacteriocin [*Lactococcus*]; WP_101913518.1, garvicin Q family class II bacteriocin [*Lactococcus lactis*]; WP_032950344.1, garvicin Q family class II bacteriocin [*Lactococcus cremoris*]; PCS20759.1, hypothetical protein RU92_GL000407 [*Lactococcus cremoris* subsp. *tructae*]; WP_153041313.1, garvicin Q family class II bacteriocin [*Lactococcus cremoris*]; PCS13338.1, hypothetical protein RU89_GL002247 [*Lactococcus cremoris*]; EQC58050.1, hypothetical protein LLT6_11035 [*Lactococcus lactis* subsp. *cremoris* TIFN6]; WP_181189605.1, garvicin Q family class II bacteriocin [*Bombilactobacillus bombi*]; WP_086269000.1, MULTISPECIES: garvicin Q family class II bacteriocin [*Enterococcus*]; WP_142434592.1, MULTISPECIES: garvicin Q family class II bacteriocin [Bacteria]; WP_195957363.1, garvicin Q family class II bacteriocin [*Enterococcus gallinarum*]; WP_144335830.1, garvicin Q family class II bacteriocin [*Enterococcus gallinarum*]; WP_021150467.1, garvicin Q family class II bacteriocin [*Enterococcus gallinarum*]; WP_146565604.1, garvicin Q family class II bacteriocin [*Streptococcus pharyngis*]; WP_116877990.1, garvicin Q family class II bacteriocin [*Streptococcus chenjunshii*]; WP_058621360.1, garvicin Q family class II bacteriocin [*Streptococcus gallolyticus*]; WP_095559356.1, garvicin Q family class II bacteriocin [*Streptococcus thermophilus*]; WP_018364975.1, garvicin Q family class II bacteriocin [*Streptococcus caballi*]; WP_074450823.1, garvicin Q family class II bacteriocin [*Streptococcus equinus*]; WP_114318989.1, garvicin Q family class II bacteriocin [*Streptococcus gallolyticus*]; WP_114318987.1, garvicin Q family class II bacteriocin [*Streptococcus gallolyticus*]; AAG29818.1 (**Bovicin 255**), bovicin 255 peptide precursor [*Streptococcus sp.* LRC 0255]; WP_074615778.1, garvicin Q family class II bacteriocin [*Streptococcus equinus*]; WP_039697853.1, garvicin Q family class II bacteriocin [*Streptococcus equinus*]; WP_015695690.1, garvicin Q family class II bacteriocin [*Streptococcus infantarius*]; AAR02622.1, bovicin 255 variant [*Streptococcus equinus*]; AAR02624.1, bovicin 255 variant [*Streptococcus equinus*]; WP_039694458.1, garvicin Q family class II bacteriocin [*Streptococcus gallolyticus*]; WP_003025420.1, MULTISPECIES: garvicin Q family class II bacteriocin [*Streptococcus*]; AGU72420.1, hypothetical protein SCRE_0568 [*Streptococcus constellatus* subsp. *pharyngis* C232]; WP_143454876.1, garvicin Q family class II bacteriocin [*Ligilactobacillus ruminis*]; WP_204784178.1, garvicin Q family class II bacteriocin [*Ligilactobacillus agilis*]; WP_170091846.1, garvicin Q family class II bacteriocin [*Ligilactobacillus agilis*]; WP_094130870.1, garvicin Q family class II bacteriocin [*Ligilactobacillus agilis*]; WP_167589585.1, garvicin Q family class II bacteriocin [*Ligilactobacillus agilis*]; WP_179923422.1, garvicin Q family class II bacteriocin [*Streptococcus danieliae*]; WP_089135223.1, garvicin Q family class II bacteriocin [*Ligilactobacillus murinus*]; WP_163587678.1, garvicin Q family class II bacteriocin [*Ligilactobacillus murinus*]; WP_119448348.1, garvicin Q family class II bacteriocin [*Ligilactobacillus murinus*]; WP_081610258.1, garvicin Q family class II bacteriocin [*Ligilactobacillus murinus*]; WP_076150142.1, garvicin Q family class II bacteriocin [*Ligilactobacillus murinus*]; HBV49078.1, TPA: ComC/BlpC family peptide pheromone/bacteriocin [*Lactobacillus sp.*]; WP_081579535.1, garvicin Q family class II bacteriocin [*Leuconostoc carnosum*]; WP_048486328.1, garvicin Q family class II bacteriocin [*Lacticaseibacillus rhamnosus*]; WP_016369810.1, garvicin Q family class II bacteriocin [*Lacticaseibacillus paracasei*]; EDY98021.1, hypothetical protein LRH_09920 [*Lacticaseibacillus rhamnosus* HN001]; WP_109990520.1, garvicin Q family class II bacteriocin [*Lacticaseibacillus paracasei*]; EPC50098.1 (**BacSJ**), bacteriocin BacSJ2-8 [*Lacticaseibacillus paracasei* subsp. *paracasei* CNCM I-4270]; BAB86318.1 (**Acidocin M**), acidocin M, partial (plasmid) [*Lactobacillus acidophilus*]; WP_204126200.1, garvicin Q family class II bacteriocin [*Lacticaseibacillus paracasei*]; WP_002264120.1, garvicin Q family class II bacteriocin [*Streptococcus mutans*]; WP_019314526.1, garvicin Q family class II bacteriocin [*Streptococcus mutans*]; WP_019313835.1, garvicin Q family class II bacteriocin [*Streptococcus mutans*]; WP_002262918.1, garvicin Q family class II bacteriocin [*Streptococcus mutans*]; WP_002267385.1, garvicin Q family class II bacteriocin [*Streptococcus mutans*]; QFG40385.1, bacteriocin class II with double-glycine leader peptide family protein [*Streptococcus mutans*]; WP_002282764.1, garvicin Q family class II bacteriocin [*Streptococcus mutans*]; WP_002312350.1, garvicin Q family class II bacteriocin [*Streptococcus mutans*]; WP_002310902.1, garvicin Q family class II bacteriocin [*Streptococcus mutans*]; WP_019802917.1, garvicin Q family class II bacteriocin [*Streptococcus mutans*]; WP_002286438.1, garvicin Q family class II bacteriocin [*Streptococcus mutans*]; WP_002270875.1, garvicin Q family class II bacteriocin [*Streptococcus mutans*]; WP_002295719.1, garvicin Q family class II bacteriocin [*Streptococcus mutans*]; WP_002307353.1, garvicin Q family class II bacteriocin [*Streptococcus mutans*]; WP_128833820.1, garvicin Q family class II bacteriocin [*Streptococcus troglodytae*]; ESS18785.1,

putative bacteriocin peptide [*Streptococcus mutans* PKUSS-HG01]; ESS18432.1, putative bacteriocin peptide [*Streptococcus mutans* PKUSS-LG01]; WP_003081942.1, garvicin Q family class II bacteriocin [*Streptococcus macacae*].
